# Heterogeneity in 10-year course trajectories of moderate to severe major depressive disorder among veterans

**DOI:** 10.1017/S2045796024000465

**Published:** 2024-11-13

**Authors:** V. Panaite, S. L. Luther, D. K. Finch, N. J. Cohen, S. K. Schultz, A. Tsalatsanis, P. N. Pfeiffer

**Affiliations:** 1 Research & Development Service, James A. Haley Veterans’ Hospital, Tampa, FL, USA; 2 Department of Psychiatry and Behavioral Neurosciences, University of South Florida, Tampa, FL, USA; 3 College of Public Health, University of South Florida, Tampa, FL, USA; 4 Department of Environmental Medicine and Public Health, Icahn School of Medicine at Mount Sinai, New York, NY, USA; 5 Mental Health and Behavioral Sciences, James A. Haley Veterans’ Hospital, Tampa, FL, USA; 6 VA Center for Clinical Management Research, VA Ann Arbor Healthcare System, Ann Arbor, MI, USA; 7 Department of Psychiatry, University of Michigan Medical School, Ann Arbor, MI, USA

**Keywords:** Depression, trajectories, mental health, Veterans, healthcare

## Abstract

**Aims:**

Epidemiological studies show that despite the episodic nature, the long-term trajectory of depression can be variable. This study evaluated the heterogeneity of 10-year trajectory of major depressive disorder (MDD) related service utilization and associated clinical characteristics among US Veterans with a first diagnosis after 9/11.

**Methods:**

Using a cohort design, electronic health record data for 293,265 Operation Enduring Freedom and Iraqi Freedom (OEF/OIF) Veterans were extracted to identify those with MDD between 2001 and 2021 with a full preceding year of clinical data and 10 years following the diagnosis. Latent class growth analysis compared clinical characteristics associated with four depression trajectories. Across all Veterans Affairs (VA)hospitals, 25,307 Veterans met our inclusion criteria. Demographic and clinical information from medical records was extracted and used as predictors of depression 10-year trajectories.

**Results:**

Among the study cohort (*N* = 25,307), 27.7% were characterized by brief contact, 41.7% were later re-entry, 17.6% were persistent contact and 12.9% were prolonged initial contact for depression related services. Compared to Veterans with trajectories showing brief contact, those with protracted treatment (persistent or prolonged initial contact) were more likely to be diagnosed with comorbid posttraumatic stress disorder (PTSD) and with MDD that was moderate to severe or recurrent.

**Conclusions:**

Depression is associated with a range of treatment trajectories. The persistent and prolonged initial contact trajectories may have distinct characteristics and uniquely high resource utilization and disability income. We can anticipate that patients with comorbid PTSD may need longer-term care which has implications for brief models of care.

## Introduction

Most adults experience depression as episodic (Kanter *et al.*, 2008). Epidemiological studies have shown that despite the episodic nature, the long-term trajectory of depression can be variable (Eaton *et al.*, 2008; Kanter *et al.*, 2008; Musliner *et al.*, 2016). In a 23-year study of outcomes after a first episode of depression among US adults, Eaton and colleagues (2008) found that nearly 50% of people experienced one major depressive episode, 35% experienced multiple distinct episodes and a far smaller group experienced depression as chronic. The episodic nature and long-term variability in trajectories of depression leads to a constant flux in population and individual needs for mental health services. A recent study (Musliner *et al.*, 2016) evaluated the heterogeneity of major depressive disorder (MDD) diagnoses in the Danish Psychiatric Central Research Register, which collects data from treatment points of contact. In this study, Musliner and colleagues (Musliner *et al.*, 2016) followed a cohort of patients for 10 years after their first depression diagnosis in their medical charts and used group-based trajectory models to identify subgroups within the population and patient characteristics associated with subgroup membership. Generally, female sex and severe depression at initial diagnosis were associated with protracted care trajectories (Musliner *et al.*, 2016). This information has implications for both patients and systems. At the patient level, treatment efforts can be planned accordingly. At the system level, this knowledge could inform the planning of future efforts to further develop the mental health field to meet patient needs (Cuijpers *et al.*, 2013; Kazdin and Blase, 2011; Kazdin and Rabbitt, 2013).

Much of the knowledge we have regarding depression trajectories comes from studies on civilian populations. Depression is highly prevalent among Veterans who show greater health services use and productivity loss due to depression relative to civilians (Zhdanava *et al.*, 2021). The few studies on military personnel or Veterans have focused on self-reported depression symptom trajectories (Armenta *et al.*, 2019; Karstoft *et al.*, 2020; Sampson *et al.*, 2022). These studies used diverse methods and designs, such as dynamic cohort study design (Sampson *et al.*, 2022), variable timepoints (Karstoft *et al.*, 2020) and highly disparate timepoints (3–4 years apart; Armenta *et al.*, 2019; Karstoft *et al.*, 2020), leading to disparate findings. To our knowledge, our study is the first to evaluate trajectories of depression diagnoses over a 10-year period within the context of health service utilization among Veterans using medical record data. We applied the Musliner and colleagues’ analytic framework (Musliner *et al.*, 2016) to a comparatively large cohort of Veterans, using a similar methods and design which allowed to compare and contrast findings. The main goal of this study was to examine the heterogeneity in 10-year trajectories of MDD in a Veteran cohort starting from their first depression diagnosis. Furthermore, we were interested to describe the clinical and demographic characteristics associated with the different depression trajectories.

## Methods

### Data sources and study sample

We obtained data from the VA Corporate Data Warehouse through the Veterans Affairs Informatics and Computing Infrastructure (VINCI), which contains data from electronic medical records of all Veterans Health Administration (VHA)patients. Inclusion criteria were as follows: Veterans from Operation Enduring Freedom and Iraqi Freedom (OEF/OIF) cohort with VA services received between 2001 and 2021, with at least one depression diagnosis and one positive depression screen Patient Health Questionnaire (i.e., PHQ-2) at any time in the study period (*N* = 293,265), with a full year of data available prior to the first depression diagnosis for a comparable baseline period, and with at least 10 years of VHA services to allow for a comparable number of data time points. Veterans were excluded based on the presence of diagnoses that would result in divergent care: dementia, bipolar, schizophrenia and other primary psychotic disorders (e.g., schizoaffective). The final sample was a cohort of 25,307 Veterans. Correlates comparable to those tested in the study by Musliner and colleagues (Musliner *et al.*, 2016) were included: gender, age at first depression diagnosis, severity of initial depression diagnosis (mild or other as a comparison group, moderate, severe without psychotic features, severe with psychotic features [i.e., hallucination, delusions] based on diagnostic specifiers). In addition to these correlates, we explored race, ethnicity, marital status, rural vs. urban residence, receipt of VA benefits, the presence of prior mental health treatment and past-year diagnosis of posttraumatic stress disorder (PTSD).

### Statistical analysis

#### Trajectory groups

Latent class growth analysis (LCGA) (Jung and Wickrama, 2008; Muthén, 2004; Nagin, 1999) was used to generate group trajectories. LCGA uses maximum likelihood estimates to identify groups of patients with similar trajectories and assigns each patient a probability of membership in each group (Kertesz *et al.*, 2012; Nagin and Nagin, 2005).

The response variable for the LCGA models was a past-year contact within the VA system with an MDD diagnosis. A logistic response model was used to model the conditional distribution of the response variable in the LCGA. To identify the optimal number of trajectory groups, we followed the methods reported by Musliner (Musliner *et al.*, 2016). Briefly, we fit trajectory models with one to seven groups using a linear order term for the first group and quartic polynomial order terms for the rest of the groups. When a polynomial term in a group was not statistically significant, we refit the model with a lower order term for the particular group and repeat until all terms were significant. The trajectory models with all the polynomial terms statistically significant were evaluated based on a combination of measures that included the Bayesian information criterion (BIC), entropy, maximum average posterior probability (APP) of membership and visual inspection of the trajectory curves.

The evaluation measures for the LCGA models with one to seven latent groups are summarized in Table 1. Based on BIC, the models with four to seven groups have a better fit than models with one to three groups. Among the models with four to seven groups, the model with four groups has the highest entropy and maximum APP membership (Table 1). Thus, the rest of the analysis is performed on the model with four groups.

**Table 1.** Fit statistics for LCGA models with one to seven groups

				Proportion of population in each group^a^							
Groups	BIC	Entropy	Max APP	1	2	3	4	5	6	7	Polynomial order term per group
1	−156,945.2	.	1	100.0%							1
2	−132,377.5	0.839	0.96	65.6%	34.4%						1 4
3	−124,083	0.815	0.95	34.9%	39.9%	25.2%					1 4 4
**4**	**−121,077.3**	**0.845**	**0.95**	**27.8%**	**41.7%**	**17.6%**	**12.8%**				1 4 4 4
5	−118,843.2	0.77	0.91	23.7%	33.8%	14.9%	14.0%	13.6%			1 4 4 4 4
6	−118,284.1	0.731	0.89	10.9%	35.2%	20.1%	10.7%	11.3%	11.7%		0 2 3 4 4 2
7	−117,780.1	0.776	0.91	9.4%	10.2%	18.1%	5.4%	4.9%	10.4%	41.7%	1 4 3 4 4 2 3

a Group membership is calculated based on posterior probabilities.

Univariate multinomial logistic regression (MLR) models with dependent variable the group trajectory derived by the LCGA were used to calculate and report the odds and 95% confidence interval (CI) of a patient to belong in a group as opposed to a reference group. All analysis was performed in Stata (StataCorp, 2021) and trajectories were modelled using the traj package (Jones and Nagin, 2007, 2013; Jones *et al.*, 2001). Significance level was set at *p* < 0.05.

## Results

### Patient population

We identified 25,307 patients meeting the study’s inclusion criteria. Among these patients, 25,252 (99%) had no missing values in the variables of interest and were included in the analysis. Eighty-four percent of the population (21,280) were male, and the mean (SD) age at the first recorded MDD diagnosis was 34.1 (9.25) years. A total of 9,021 (35.7%) patients were diagnosed with PTSD at baseline, 848 (3.4%) patients were not receiving VA benefits, 747 (3%) had a history of combination treatment of psychotherapy and medication in the past prior to their initial depression diagnosis and 474 (1.9%) had severe MDD at their first episode. Detailed demographic and clinical characteristics of patient population are described in Table 2.

**Table 2.** Demographics and clinical characteristic of the study and groups

	Overall	Brief contact	Later re-entry	Persistent contact	Prolonged initial contact
*N*^a^	25,252	7536 (29.8%)	10,142 (40.2%)	4481 (17.7%)	3093 (12.2%)
Age mean (SD)	34.1 (9.25)	33.5 (9.2)	33.8 (9.07)	36.2 (9.46)	33.9 (9.23)
Female *N* (%)	3972 (15.7%)	839 (11.1%)	1625 (16%)	968 (17.5%)	540 (17.5%)
Race *N* (%)					
White	17,630 (69.8%)	5340 (70.9%)	7098 (70%)	3066 (68.4%)	2126 (68.7%)
Black or African American	5196 (20.6%)	1450 (19.2%)	2100 (20.7%)	1005 (22.4%)	641 (20.7%)
Other	2426 (9.6%)	746 (9.9%)	944 (9.3%)	410 (9.1%)	326 (10.5%)
Ethnicity *N* (%)					
Hispanic or Latino	3213 (12.7%)	859 (11.4%)	1238 (12.2%)	714 (15.9%)	402 (13.0%)
No Hispanic or Latino	21,673 (85.8%)	6522 (86.5%)	8774 (86.5%)	3725 (83.1%)	2652 (85.7%)
Marital status *N* (%)					
Divorced	5419 (21.5%)	1574 (20.9%)	2121 (20.9%)	1026 (22.9%)	698 (22.6%)
Married	12,938 (51.2%)	3863 (51.3%)	5166 (50.9%)	2384 (53.2%)	1525 (49.3%)
Never married	5463 (21.6%)	1698 (22.5%)	2252 (22.2%)	831 (18.5%)	682 (22%)
Separated	1256 (5%)	361 (4.8%)	529 (5.2%)	204 (4.6%)	162 (5.2%)
Single	37 (0.1%)	8 (0.1%)	16 (0.2%)	6 (0.1%)	7 (0.2%)
Widow/widower	3 (0%)	1 (0%)	1 (0%)	1 (0%)	0 (0%)
Widowed	136 (0.5%)	31 (0.4%)	57 (0.6%)	29 (0.6%)	19 (0.6%)
Rural *N* (%)	3431 (13.6%)	768 (10.2%)	1596 (15.7%)	711 (15.9%)	356 (11.5%)
NSC *N* (%)	848 (3.4%)	322 (4.3%)	387 (3.8%)	55 (1.2%)	84 (2.7%)
PTSD *N* (%)	9021 (35.7%)	2751 (36.5%)	3502 (34.5%)	1486 (33.2%)	1282 (41.4%)
Tx 365 days before diagnosis *N* (%)					
No treatment	20,116 (79.7%)	6034 (80.1%)	8121 (80.1%)	3522 (78.6%)	2439 (78.9%)
Only psychotherapy	763 (3%)	257 (3.4%)	275 (2.7%)	131 (2.9%)	100 (3.2%)
Only medication	3626 (14.4%)	1031 (13.7%)	1476 (14.6%)	669 (14.9%)	450 (14.5%)
Both	747 (3%)	214 (2.8%)	270 (2.7%)	159 (3.5%)	104 (3.4%)
Recurrent depression episode *N* (%)	3320 (13.1%)	543 (7.2%)	709 (7%)	1149 (25.6%)	3320 (13.1%)
Index episode severity *N* (%)					
Mild/unspecified	22,589 (89.5%)	7108 (94.3%)	9559 (94.2%)	3588 (80.1%)	2342 (75.8%)
Moderate	1981 (7.8%)	324 (4.3%)	455 (4.5%)	643 (14.3%)	559 (18.1%)
Severe	474 (1.9%)	72 (1%)	100 (1%)	175 (3.9%)	127 (4.1%)
Psychotic	208 (0.8%)	32 (0.4%)	37 (0.4%)	75 (1.7%)	64 (2.1%)

a The patient counts and percentages listed are calculated based on observed group membership.

### Trajectory groups

**Figure 1.**
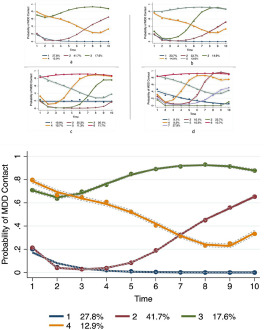
Trajectory plot of LCGA models with four to seven groups. In (a), Group 1 indicates brief contact; Group 2, later re-entry; Group 3, persistent contact; and Group 4, prolonged initial contact. The *y*-axis represents the probability of contact with the VA system with an MDD diagnosis. The *x*-axis represents the time in years after the initial MDD diagnosis. The group memberships shown are calculated based on posterior probabilities.

Figure 1a depicts the trajectory patterns of the four groups derived from the LCGA model.

Group 1 included 27.8% (computed based on posterior probabilities) of the population and is characterized by *brief contact* at the VA with an MDD diagnosis. Patients in this group had 20% probability of contact for MDD 1 year after the initial diagnosis, followed by 4% probability on year 2, declining to 0% after year 5.

The largest group was Group 2 (41.7%, computed based on posterior probabilities) and was characterized by *later re-entry* into the VA system with an MDD diagnosis. Patients in this group begun with 21% probability of MDD 1 year after the initial diagnosis, which decreased to 2% by the third year, and then steadily increased to 68% by year 10.

Group 3 (17.6%, computed based on posterior probabilities) included patients exhibiting *persistent contact* with MDD diagnosis. These patients had consistently greater than 60% (min: 64%; max: 94%) probability of an MDD diagnosis throughout the study’s timeline. After the first 2 years, these patients consistently exhibited the highest probabilities of an MDD diagnosis, including during the last 2 years, despite showing the only slight drop off in probability in years 9 and 10 among all groups.

Lastly, Group 4 (12.9%, computed based on posterior probabilities) included patients with *prolonged initial contact* at a VA facility with an MDD diagnosis. Patients in this group had high (81%) probability of contact the first year steadily declining to 20% by year 8 and increasing to 33% by year 10. Demographic and clinical characteristics of patients in each group are presented in Table 2. Note that the patient counts presented in Table 2 are based on observed group membership (i.e., the LCGA model patient allocation).

#### Patient characteristics associated with the four trajectories

Table 3 reports the results of the MLRs on the trajectory correlates in terms of odds ratios and 95% CI expressing how a correlate is associated with the observed membership of a patient in a certain group (i.e., prolonged initial contact) as opposed to a different group (i.e., brief contact).

**Table 3.** Univariate multinomial logistic regression: patient characteristics associated with trajectory type

	Later re-entry vs brief contact	Persistent contact vs brief contact	Prolonged initial contact vs brief contact	Persistent contact vs later re-entry	Prolonged initial contact vs later re-entry	Prolonged initial contact vs persistent contact
Age	1.00 (1.00–1.00)	1.03 (1.02–1.04)	1.00 (1.00–1.01)	1.03 (1.03–1.03)	1.00 (0.99–1.00)	0.98 (0.97–0.98)
Female	**1.52 (1.39**–**1.66)**	**2.20 (1.99**–**2.43)**	**1.69 (1.50**–**1.89)**	**1.44 (1.32**–**1.57)**	1.10 (0.99–1.23)	**0.77 (0.68**–**0.86)**
Race						
White	Ref	Ref	Ref	Ref	Ref	Ref
Black or African American	1.09 (1.01, 1.17)	**1.21 (1.10, 1.32)**	1.11 (0.99, 1.23)	**1.11 (1.01**–**1.20)**	1.02 (0.92–1.12)	0.92 (0.82–1.03)
Other	0.95 (0.86, 1.05)	0.96 (0.84, 1.08)	1.09 (0.95, 1.26)	1.00 (0.89–1.13)	1.15 (1.00–1.32)	1.14 (0.98–1.12)
Ethnicity						
Hispanic or Latino	Ref	Ref	Ref	Ref	Ref	Ref
Not Hispanic or Latino	0.93 (0.85, 1.02)	**0.69 (0.61, 0.76)**	**0.87 (0.76, 0.98)**	**0.74 (0.66**–**0.81)**	0.93 (0.82–1.05)	**1.26 (1.11**–**1.44)**
Marital status						
Married	Ref	Ref	Ref	Ref	Ref	Ref
Divorced/separated	1.02 (0.95, 1.10)	1.03 (0.94, 1.12)	**1.13 (1.01, 1.24)**	1.01 (0.92–1.10)	1.10 (0.99–1.21)	1.09 (0.98–1.22)
Never married	0.99 (0.92, 1.07)	**0.79 (0.72, 0.87)**	1.02 (0.91, 1.13)	**0.80 (0.73**–**0.88)**	1.02 (0.92–1.13)	**1.28 (1.13**–**1.44)**
Other	1.38 (0.94, 2.03)	1.46 (0.93, 2.29)	1.64 (1.00, 2.71)	1.05 (0.71–1.57)	1.19 (0.76–1.87)	1.13 (0.67–1.88)
Rural residence	**1.65 (1.50**–**1.80)**	**1.66 (1.49**–**1.85)**	1.14 (1.00–1.31)	1.00 (0.91–0.67)	**0.69 (0.61**–**0.78)**	**0.69 (0.60**–**0.79)**
NSC	0.89 (0.76–1.03)	**0.28 (0.21**–**0.37)**	**0.63 (0.49**–**0.80)**	**0.31 (0.23**–**0.41)**	**0.70 (0.55**–**0.89)**	**2.24 (1.59**–**3.16)**
PTSD	0.92 (0.86–0.98)	**0.86 (0.80**–**0.93)**	**1.23 (1.13**–**1.34)**	0.94 (0.87–1.01)	**1.34 (1.23**–**1.46)**	**1.42 (1.30**–**1.56)**
Tx before diagnosis						
No treatment	Ref	Ref	Ref	Ref	Ref	Ref
Only psychotherapy	**0.79 (0.67**–**0.94)**	0.87 (0.70–1.08)	0.96 (0.76–1.21)	1.09 (0.89–1.36)	1.21 (0.95–1.53)	1.10 (0.84–1.43)
Only medication	1.06 (0.97–1.15)	1.11 (1.00–1.23)	1.08 (0.95–1.22)	1.04 (0.95–1.15)	1.02 (0.91–1.13)	0.97 (0.85–1.10)
Both	0.94 (0.79–1.12)	**1.27 (1.03**–**1.57)**	1.20 (0.94–1.52)	**1.36 (1.11**–**1.66)**	**1.28 (1.02**–**1.61)**	0.94 (0.73–1.22)
Recurrent index episode	0.97 (0.86–1.08)	**4.44 (3.97**–**4.95)**	**5.44 (4.84**–**6.11)**	**4.58 (4.14**–**5.07)**	**5.62 (5.04**–**6.26)**	**1.22 (1.10**–**1.36)**
Index episode severity						
Mild/unspecified	Ref	Ref	Ref	Ref	Ref	Ref
Moderate	1.04 (0.90–1.21)	**3.39 (3.41**–**4.52)**	**5.23 (4.52**–**6.05)**	**3.76 (3.31**–**4.26)**	**5.00 (4.38**–**5.71)**	**1.33 (1.17**–**1.51)**
Severe	1.03 (0.76–1.40)	**4.81 (3.64**–**6.62)**	**5.35 (3.99**–**7.17)**	**4.65 (3.63**–**5.97)**	**5.17 (3.97**–**6.75)**	1.11 (0.88–1.40)
Psychotic	0.86 (0.53–1.38)	**4.64 (3.06**–**7.04)**	**6.07 (3.96**–**9.30)**	**5.39 (3.63**–**8.01)**	**7.05 (4.69**–**10.6)**	1.31 (0.93–1.83)

Bold ORs and 95% CI indicate ORs that are below 0.90 or above 1.10 and 95% CI that does not include OR = 1.00. Interpretation of odds ratios: Odds ratios show how a variable is associated with the odds of a patient to belong to a group vs a reference group. For example, the odds 1.11 (Black or African American in the comparison of prolonged contact vs brief contact) indicate that Black or African American patients have 11% greater odds than White patients of membership in prolonged contact than brief contact.

Patient sociodemographic characteristics of the brief contact group included a greater likelihood compared to other groups of being male, white, never married, residing in urban or suburban areas and without VA benefits. Brief contact patients were more likely to have a history of psychotherapy than later re-entry patients and less likely to have been on both antidepressants and psychotherapy than persistent contact patients. Brief contact patients were much less likely to have either an initial MDD diagnosis qualified as recurrent or moderate to severe than persistent and prolonged initial contact although brief contact patients were more likely to have a first MDD diagnosis with psychotic features than later re-entry.

Patients in the later re-entry group were similar to brief contact patients except late re-entry patients were more likely to be female, from rural areas and less likely to have received psychotherapy.

Patients in the persistent contact group compared to all other groups were most likely to be female or part of a minority group, less likely to be never married and more likely to have VA benefits. Persistent contact patients were more likely to have a history of both antidepressants and psychotherapy and an initial MDD diagnosis qualified as recurrent, moderate or severe (with or without psychotic features) relative to both brief contact and later re-entry.

Finally, prolonged initial contact patients compared to all other groups were most likely to have a PTSD diagnosis comorbidity and more likely to have a recurrent or moderate first MDD diagnosis. They were the most likely to have a severe MDD initial diagnosis but only relative to brief and later re-entry groups.

## Discussion

Depression in Veterans is associated with worse outcomes for both health and mental disorders (McCarthy *et al.*, 2015; Trivedi *et al.*, 2015; Vance *et al.*, 2019), in part due to high rates of complicating comorbidities, such as PTSD. In the current study, we evaluated the heterogeneity of 10-year trajectories of MDD and associated treatment utilization. Our models following Musliner and colleagues’ (Musliner *et al.*, 2016) analytical framework resulted in the same four classes of patients: those with brief contact, patients with prolonged initial contact, those with later re-entry and patients with persistent contact with services for depression. Although we found a similar set of classes, the distribution of patients across classes differed substantially from the Danish cohort, with higher representation of more prolonged contact among Veterans.

Our distribution of trajectory classes and characteristics associated with each trajectory showed both similarities and differences from prior work. Similar to prior work (Musliner *et al.*, 2016), female sex, mental health comorbidities and higher severity of initial MDD diagnosis were all associated with longer-term service use trajectories in the current study. However, nearly three quarters of our patients showed trajectories defined by more prolonged care, either initially, later or persistently. This is in stark contrast to the cohort presented in Musliner and colleagues (Musliner *et al.*, 2016) which was nearly 80% in the brief contact class. This was surprising given that prevalence of mild severity at initial diagnosis was more than triple in our sample than in this prior work. However, our sample differs from prior samples (Eaton *et al.*, 2008; Musliner *et al.*, 2016) in a couple of significant ways. First, our sample was only 15% female, while in the civilian population, the rate of depression continues to be 2:1 females to males. Veteran men in particular may underreport symptom severity at initial diagnosis of depression to keep with military culture of emotional control (Nash *et al.*, 2009). Our sample was mainly outpatient relative to the Musliner and colleagues (Musliner *et al.*, 2016) which was a cohort of patients from a psychiatric hospital who may not have engaged in outpatient care following discharge, thus potentially explaining the combination of more severe MDD with brief contact.

Our findings may also reflect the differential needs of Veteran populations of patients who tend to show higher rates of generally more persistent mental health needs over time relative to civilians (Hundt *et al.*, 2014; Liu *et al.*, 2019). Several factors likely contribute to these findings. Comorbid PTSD is often associated with higher mental healthcare needs (Switzer *et al.*, 1999). Veterans may also have higher physical care needs associated with chronic illness which in turn are associated with depression (Vance *et al.*, 2019). It is likely that the Danish sample may experience higher general quality of life and well-being despite their mental health needs relative to our Veteran population, given prior findings that Danish adults reported higher levels of satisfaction than US respondents, even when reporting low income (Biswas-Diener *et al.*, 2010).

The prominent return to care among Veterans – nearly half of our cohort – relative to the Danish cohort which was only 7% later re-entry (Musliner *et al.*, 2016) suggests Veterans may also be more likely to develop new or worsening comorbid mental and physical conditions (McCarthy *et al.*, 2015; Trivedi *et al.*, 2015; Vance *et al.*, 2019) that can trigger a recurrence of depression. In a recent parent study on a larger cohort with at least 2 years in the VA, we found that nearly one in four Veterans received fewer than four psychotherapy sessions or less than 84 days on an antidepressant – both of which represent less than a clinically effective course of treatment and could result in increased rates of late re-entry to care (Panaite *et al.*, 2024).

Several patient characteristics predicted treatment trajectories. We found men were less likely than women to return to care after initial treatment. This could represent greater overall less treatment-seeking among men compared to women, although women may also be more likely to return to care if they have greater rates of recurrence of depression. Patients who did not receive psychotherapy in the past were also more likely to return to care, which is consistent with prior studies showing psychotherapy may have longer-term benefits than treatment with antidepressant medications (Guidi and Fava, 2021). Finally, patient characteristics associated with lower probabilities of return to care mirrored characteristics associated with drop out of care in a larger cohort (Panaite *et al.*, 2024): male gender and prior psychotherapy were associated with underutilization of mental health services.

Current findings should be interpreted within the context of some limitations. For example, trajectories are based entirely on presence of diagnosis within medical charts and provider rated severity, without patient reported symptom severity measures, such as the PHQ-9 which was not administered systematically during the study period (e.g., Panaite *et al.*, 2019). In the current study, we focused on unipolar depressive disorders for two reasons: one, unipolar depression is highly heterogeneous as a syndrome and including additional diagnoses that share the presence of major depressive episodes (e.g., schizoaffective disorder, bipolar disorder) but diverge in other important areas such as genetic loading, prevalence and disease trajectories, would complicate generalizing the findings to the most common occurrences of depression; second, we found it important to be able to compare and contrast findings with a prior study that used similar criteria (Musliner *et al.*, 2016). Future studies should also use structured and semi structured clinical interviews for formal diagnosis or, alternatively, use validated self-report measures that assess symptoms for a probable/provisional diagnosis.

Current findings provide a baseline for understanding the mental healthcare history of a cohort of Veterans involved in military operations after 9/11. Understanding this cohort’s initial trajectory of mental healthcare needs helps project and plan for the future needs of Veterans, such as more precisely estimating volume of return to care patients, or projected length of care. For example, the high rate of patients returning to care raises questions regarding whether these patients would most effectively be treated initially in primary care, particularly if they previously responded to primary care treatment, or if they should start with specialty care given likely recurrence despite prior treatment. Another empirical question could be clarifying whether patients should receive the same prior type of treatment (e.g., antidepressants) or switch to or add a different treatment (e.g., psychotherapy). A recent meta-analysis including 17 randomized clinical trials of 2283 participants showed that the sequential integration of psychotherapy following response to acute-phase pharmacotherapy, alone or combined with antidepressant medication, was associated with reduced risk of relapse and recurrence in MDD in civilians (Guidi and Fava, 2021) suggesting that specific sequences of treatments may lead to lower rates of return to care. Understanding how care is sequenced in Veterans with depression is key, especially given recent findings that those Veterans showing early improvement to antidepressants were more likely to be in the responsive trajectory groups, again, likely to show lower probability of care later on (Hicks *et al.*, 2023). Furthermore, understanding the potentially dynamic factors that could lead to drop out of care versus return to care at any given time in the trajectory would be highly impactful for our prediction of these effects. Future studies could look at variability of group trajectories across other mental disorders that can include major depressive episodes (i.e., schizoaffective disorder, bipolar), are highly comorbid (i.e., Generalized Anxiety Disorder [GAD], PTSD) and possibly share aetiology through trauma exposure (e.g., PTSD, other stress reaction diagnoses) with depression. Finally, expanding on structured data extracted from clinical charts by adding variables available in free text in clinical notes via text mining could improve our descriptive models by including more specific variables, such as reasons for drop out.

In conclusion, we found that Veterans exhibit depression trajectories similar in shape to those found in Danish patients, however membership to trajectories showing longer-term involvement in care was more prevalent among Veterans than civilian patients. Understanding the pattern of engagement with care for depression is key to planning for future mental healthcare utilization. Among our Veterans, female, minority and Veterans receiving VA benefits for military associated disabilities were more likely to engage with care for depression persistently. Healthcare systems should prioritize further understanding both reasons for return to care and drop out of care or refraining from returning to mental healthcare.

## Data Availability

Data sharing is restricted by VHA policies. Deidentified data can be shared with individual parties following appropriate VHA channels upon request. Data are not available for sharing through publicly available repositories. Materials and/or Code: Available upon request.
